# Design and Evaluation of a Solo-Resident Smart Home Testbed for Mobility Pattern Monitoring and Behavioural Assessment

**DOI:** 10.3390/s20247167

**Published:** 2020-12-14

**Authors:** Mohsen Shirali, Jose-Luis Bayo-Monton, Carlos Fernandez-Llatas, Mona Ghassemian, Vicente Traver Salcedo

**Affiliations:** 1 Computer Science and Engineering, Shahid Beheshti University, Tehran 19839-63113, Iran; 2 Process Mining 4 Health Lab–SABIEN-ITACA Institute, Universitat Politècnica de València, 46022 Valencia, Spain; jobamon@itaca.upv.es (J.-L.B.-M.); cfllatas@itaca.upv.es (C.F.-L.); vtraver@itaca.upv.es (V.T.S.); 3 Department of Clinical Sciences, Intervention and Technology (CLINTEC), Karolinska Institutet, 17177 Stockholm, Sweden; 4 BT Applied Research Labs, Adastral Park, Ipswich IP5 3RE, UK; mona.ghassemian@bt.com

**Keywords:** smart home, testbed, process mining, human mobility pattern monitoring, behaviour assessment

## Abstract

Aging population increase demands for solutions to help the solo-resident elderly live independently. Unobtrusive data collection in a smart home environment can monitor and assess elderly residents’ health state based on changes in their mobility patterns. In this paper, a smart home system testbed setup for a solo-resident house is discussed and evaluated. We use paired Passive infra-red (PIR) sensors at each entry of a house and capture the resident’s activities to model mobility patterns. We present the required testbed implementation phases, i.e., deployment, post-deployment analysis, re-deployment, and conduct behavioural data analysis to highlight the usability of collected data from a smart home. The main contribution of this work is to apply intelligence from a post-deployment process mining technique (namely, the parallel activity log inference algorithm (PALIA)) to find the best configuration for data collection in order to minimise the errors. Based on the post-deployment analysis, a re-deployment phase is performed, and results show the improvement of collected data accuracy in re-deployment phase from 81.57% to 95.53%. To complete our analysis, we apply the well-known CASAS project dataset as a reference to conduct a comparison with our collected results which shows a similar pattern. The collected data further is processed to use the level of activity of the solo-resident for a behaviour assessment.

## 1. Introduction

**Figure 1:**
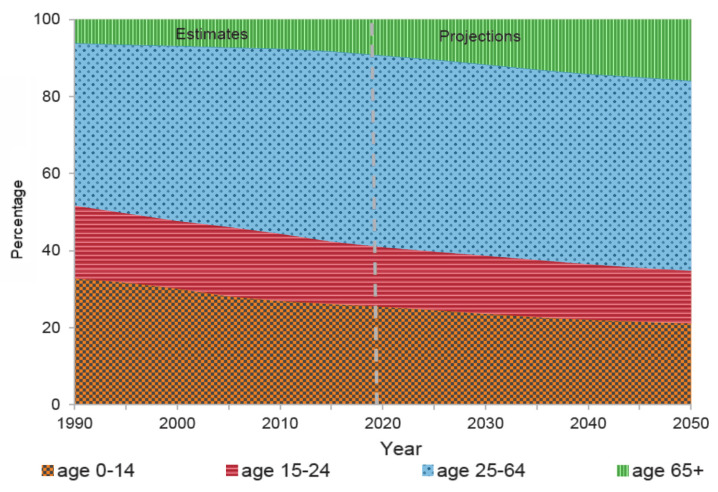
Percentage of the total population by sex and age group and the estimation of aging population growth [3].

The necessity of looking for novel solutions to provide independently living option for elderlies due to the elderly population growth and the shift in base of the age pyramid-mostly in developed countries [1], along with the scientific advances in the implementation of communication infrastructures in various aspects, puts the spotlight on smart home and their practical applications. Globally, according to statistics and reports such as [2,3], the share of the population aged 65 years or over increased from six percent in 1990 to nine percent in 2019. That proportion is projected to rise further to 16 percent by 2050 (Figure 1). So that one in six people in the world will be aged 65 years or over, which brings up more service demand for the aging population that pushes the health and wellbeing issues among the top priorities in societies worldwide [4]. Plextek survey of future health suggests that 76% of the population is concerned about their elderly relatives if experiencing an emergency such as a heart attack or fall specially when living alone [5,6].

Advances in pervasive computing and their impact on understanding human behaviour especially for the aging population need, has grown dramatically in the last decade [7]. The modelling of human behaviour provides insights into human habits and their influence on health, sustainability, and wellbeing. Human-centric technologies such as Ambient Assisted Living (AAL) technologies and smart homes can assist with health monitoring, energy efficiency, and behavioural interventions.

A smart home is made up of a network of physical devices to provide electronic, sensor, software, and connectivity within a house [8]. Sensors are installed in a house to capture readings when residents perform daily routines in order to gain insights on human activities, movements, and gestures which are generally known as human daily behaviours [7,9] to assess the functional ability of residents for living independently [10,11]. For instance, an elderly patient aftercare monitoring is a common application scenario for smart environments to observe their recovery in their own space, immediately after being discharged from hospital for a duration suggested by their physicians [12].

Three different categories are proposed for wireless AAL monitoring solutions [10,13]:

(1) Methods which are known as “vision-based” basically capture a series of images by a video or a camera to use them for monitoring the activity and mobility pattern. Despite weaknesses have limited the practical usage of vision-based methods such as suffering from background change, illumination variations, environmental noise, and ambient occlusion, but a satisfactory performance is achievable in many cases. However, when it comes to the privacy policy enforcement, the use of a camera is not welcome and considered as invasive. In addition, the high cost for hardware supply and deployment reduced the wide usage of vision based methods; hence, these methods are often preferred to be used for games, security, and safety surveillance [14].
(2) Second solution category, wearable sensor-based methods, works with wearable devices worn or carried by an elderly people. Sensors such as accelerometers, gyroscopes, GPS, magnetometers, electrocardiogram (ECG), blood pressure, and temperature are examples of the second category [7]. The quality of user experience can degrade since carrying and maintaining one or more wireless nodes all the time can become inconvenient and obtrusive.
(3) The third solution of ambient monitoring free the elderlies from having to wear additional devices [15]; hence, they can be considered as a zero-effort technology, which means the technology frees the user from the effort needed to operate the technology correctly [6]. However, there are still demands for installation of sensors such as motion detectors in the residential environment for a period of time, which in turn, can impose costs for installation and maintenance [16]. Sensors in this category can provide awareness about resident context (location, preferences, and activities), the physical context (lighting and temperature), and time context (hour of the day, day of the week, season, and year) [7]. Depending on the type of installed sensors, the intrusiveness of such solution can vary but they are able to protect the residents’ privacy.

In this work, we aim to design a testbed, based on the third category (ambient monitoring) for non-intrusive monitoring of the elderly in their residential. This enables us to set up a proof-of-concept to evaluate our generic solution for behaviour assessment based on the resident’s mobility pattern and predicting potential medical condition. Detecting elderly movements as they occur in different locations of a solo-resident home can be used to determine the activity level of the elderly, the time and duration of his activities and their variabilities during a long period of data collection to be used for health assessment. We use off-shelf inexpensive passive infra-red (PIR) motion detectors but can include other types of sensors that fulfil our motivation. The results of this study show that mobility pattern of residents can be monitored in remote using passive sensor technology, while subjects were able to carry out their designated activities without any serious obstruction from the sensors. Additionally, we outline the problems where incorrect data collection can cause for data annotation and analysis and provide a guidance on how to prevent these problems at the very beginning of smart home system design.

The rest of this paper is organised as follows: Section 2 provides an overview of a selection of appropriate solutions for a smart home testbed with respect to the sensor devices, applications of collected data and data validation methods. Section 3 describes our testbed design and data collection protocol. Section 4 provides an evaluation analysis using implementation. Section 5 discusses the use of day-level activity features to assess the health and wellbeing of a solo-resident. Finally, Section 6 concludes the paper.

## 2. Related Work

Early commercialised smart home systems had a steep learning curve and complicated device setup procedures [17] but products from well-known companies such as Samsung’s SmartThings [18], Apple’s HomeKit [19], Vera Control’s Vera3 [20], Google’s Weave/AndroidThings (Brillo) [21], TeleAlram [22], Birdie [23], and AllSeen Alliance’s AllJoyn [24] are examples of cloud-based user-friendly systems which provide a programming framework for third-party developers to build further applications [25]. Furthermore, a number of smart environment research platforms and testbeds have been implemented [17] and the resulting datasets are available for researchers to mine, including the CASAS project [26], the TigerPlace [27], the MavHome project [28], the Gator Tech Smart House [29], the iDorm [30], and the Georgia Tech Aware Home [31] as well as projects which are ongoing such as Technology Integrated Health Management (TIHM) [32].

The residents’ location is a first factor for context-aware service provisioning which is required for many in-home applications such as home entertainment, automatic device control, healthcare systems, activity of daily life (ADL) recognition [15], elderly monitoring, and child monitoring [33]. Ease of use (by considering user’s point of view) and acceptable performance (from the expert viewpoint) should be two main features of a desirable smart home system in order to find location. In general, the parameters such as user comfort, user privacy, and the role of user in data collection (i.e. carrying or wearing a device) have significant effects at achieving user experience. Furthermore, other determinants including the accuracy of selected technology, the required equipment, installation, and cost are involved in decision about the most practical technologies [33].

While the use of cameras and microphones provide rich information about user activities, the use of such sensors is intrusive and effects on user’s behaviour and experience [34]. Rashidi et al. [17] reported their observation based on 20 participants to be uniformly reluctant to allow video data or to wearables and rather preferred the installed sensors in a smart environment. Employing networks of PIR motion detectors leads to an inexpensive, scalable and reliable solution which detects presence and movement of heat sources (body temperature) and a proof of concept about the capability of employing PIR sensors to provide useful contextual information is presented in [35]. The PIR sensors and plug-in power meters have been investigated before in [34,36] to see how they can be used for activity recognition purposes and utility measurements; however, the privacy issues are not addressed.

In addition to these technology-related parameters, due to the lack of rich physical datasets to test the algorithms, the performance of smart environment technologies is difficult to assess as well. To create robust, usable smart environment technologies, generating and disseminating smart home datasets is very important. Generating datasets and making them available to the public [37] improve collaborative technology evaluation. As a step forward, we also aim to contribute to the research society by publicly availing our collected datasets upon publication.

Many challenges during data collection have led to this lack of rich datasets including cleaning data, the data annotation, and generation of sufficiently varied data. The need for sufficient varied data caused due to the inherent nature of human behaviour. Performing an activity by a human is typically linked with uncertainty, variety, concurrency, and overlap. Furthermore, people mostly have different ways for doing an activity or even an individual can use various ways or different places and times to fulfil the same activity which are known as the case of “inter-subject variation” and “intra-subject variation”, respectively [14]. Discontinuous varied-order mining method (DVSM) [17] is one of the solutions which was proposed to detect frequent patterns with possible discontinuity and variability in the ordering to address the intra-subject variability issue such as different eating style in an individual over time. By discovering the common activity patterns for everyone instead of using pre-selected activities, DVSM could also address the issue of inter-subject variability such as different eating styles in individuals. The “Bag of Sensor Events” strategy is another novel feature engineering approach which is introduced in [38] to address the challenge of performing different actions in different order. The uncertainty in doing the activities could be decreased by taking the advantages of the combination of captured data via different sensors located in different places. This approach considers the frequency of each sensor event occurrence regardless of the order of sensor events to address the variation of a specific individual behaviour and also used different behavioural patterns of smart home’s inhabitant to distinguish the residents.

Apart from having enough data, there are also challenges in the data annotation. Data annotation is done manually most of the time and there are many approaches available, such as participant self-annotation (to note each activity manually after doing it [17]) or perform a pre-defined set of activities (in this way, the correct labels are determined before data collection [17]). Furthermore, annotating the data by analysing the sensor data or hand labelling from raw sensor data is a tedious task and subject to annotation errors. Therefore, none of the annotation solutions are practical for the case of deployment in elderly homes [37]. When the elderly with dementia are supposed to be the user of an activity monitoring system, even the expectation of remembering what they did is too high; hence, it is unreasonable to expect them to accurately record activities their activities and times [17]. On this point, the PIR sensors can also be used for annotating the data in smart homes, but challenges and considerations should be noticed; hence, we look at this capability in more depth.

A PIR system as an indoor location system has detection errors that can affect the annotating process. The distribution of the sensors can affect the quality of the detections when two or more sensors can detect the resident at the same time. To detect such situations, a global view of the resident paths is needed in a human-readable format. In that line, a process mining technique [39] is a paradigm that comes from business process management and is useful to extract the information about the process. Applying different discovery algorithms to the event log of the location system, a graphical representation of the process model or pathway can be obtained. Moreover, process mining explains the underlying information of the process. The process mining algorithm, namely, the parallel activity log inference algorithm (PALIA), is one of the algorithms which has been used successfully combined with Indoor Locations Systems to analyse movements of people, as for example using RFID sensors in a hospital to track nurses’ processes [40] or using Bluetooth beacons in a shopping mall to analyse gender behaviours of buyers [41]. PALIA uses different syntactical pattern recognition techniques to generate a readable model of the process in the form of a formal automaton called timed parallel automaton (TPA) [42]. To use it, the PMApp tool provides functionalities to load the datasets, pre-process it, realise the discovery of the model using PALIA, represent it as a TPA and finally allows to apply different enhancement techniques to highlight information (heat maps, statistics, charts, etc.). The detection of the process has different applications as we see in the referenced articles, but in our case of study, this tool can be useful to validate the correctness of the location system detections and at the end for data annotation.

To sum up and according to the approaches proposed within literature, a desirable smart home solution should be able to determine when the activities occur and then must be able to perform analysis on their timing to determine long-term trends and assess activity variability. Moreover, intelligent algorithms can help the caregivers to measure how regular and consistent the inhabitants can complete their usual routines [17,43]. Such capability can lead to monitoring of functional health by tracking the occurrence of regular activities and detecting changes in an individual’s lifestyle. However, the data collection in smart homes can be associated with serious challenges as reviewed; hence, the required system for data collection should be carefully designed to meet the system goals and at the same time minimise these challenges in order to take most out of smart homes. The design of such a smart home system and its considerations is an issue which needs to be examined much more and for this reason we describe our proposed approach in the following of this paper.

## 3. Smart Home Testbed Design

In this section, we describe our solo-resident household testbed and the data collection protocol in different parts. First, we explain the reasons for choosing PIR sensors to be used without the need for other types of sensors as our data collection devices. Then, sensor installation method in our testbed implementation is described in the following.

### 3.1. Sensor Selection

Before using sensors in any setting, there are practical and ethical issues that have to be evaluated. Practical issues that need to be identified include types of sensors and types of collected information [27]. As mentioned earlier in Section 2, to monitor human activity, sensors can be worn by the resident, cameras can monitor mobility obtrusively, or ambient sensors can be placed unobtrusively in an apartment. Consequently, choices made about the types of sensors and information captured raises several ethical considerations. Privacy and willingness of elderlies to adopt smart home technology greatly concerns developers and researchers interested in this type of instrumentation. Somehow, privacy defined as an ability to control the access to personal information; therefore, the willingness of people to live in a smart setting and the acceptance of certain types of smart home technologies depends on how to keep their independency and control of their lifestyles [27]. In such situations, people are more inclined to use equipment which collect less data from their environment and activities as well as not limit their freedom to move and convenience at their own places, but at the same time, the collected data should provide reliable service to them. Hence, it can be concluded that passive sensors can be more acceptable choices to be installed in smart environments.

In addition, it should be considered that the target scenario of our designed system is aftercare monitoring of elderly patients. So, on the contrary of demonstrations of smart homes and new buildings with embedded smart technologies, this system should be installed in any home in a plug-and-play level of ease. Based on the recommendations provided by a study in 2019 towards the broader adoption of smart home technology [44], a fundamental step to reassure the acceptance of smart home technologies is to minimise the potential disturbance of installation. The appearance and visual aspects of the home are so important for inhabitants and raised concerns of people about their home decoration during the installation of equipment should be considered [44]. The rough sensors and cluttering the house space with long wires and computers will be not accepted [27]. Thus, using small, wireless sensors with little minimum effort for installing and maintaining them (e.g., replacing batteries if needed) are key factors to be considered when recommending such systems.

To address the target scenario of this paper by developing a non-intrusive and non-obtrusive approach and minimising the required cost for set up and maintenance, low-cost PIR off-shelf motion sensors are selected to be used in a similar setup to [27,35] for smart home analysis. These sensors detect presence and movement of heat sources without giving residence a sense of being watched. Furthermore, PIR sensors are small and lightweight to be mounted on the wall or ceiling with double-sided foam adhesive. On the other hand, the smart home system should consider restricted resources of sensors for data usability and computational overhead cost. At the end, by considering all of these aspects, a smart home solution designed based on using PIR sensors.

### 3.2. The Proposed Solution for Data Collection and Analysis

For data collection purpose, the environment is equipped with connected PIR sensors to generate readings with an added timestamp to record sequences of resident’s daily activity. The PIR sensors are mounted in such a way that a sensor is located in each individual area of the house and their sensing angles are adjusted to cover the entrance of area as well. In addition, sensors are paired so that passing each doorway of a house will inevitably lead to the capturing of two events: one due to the triggering of the corresponding sensor within the room and one by the sensor in the adjacent area. Hence, if we consider two sensors that are installed in two adjacent areas as a pair, whenever two consecutive events of these two pairs of sensors are observed in the recorded data, it indicates passing of the doorway and the direction of movement (entering or leaving the area) can be also determined according to the sequence of these two recorded events. In other words, when a person enters any area/room in the home, an event is recorded by the corresponding sensor within that area and transmitted to the base (sink) node. These capturing and transmitting packets by the sensor will continue as long as the person moves in that area. Further, by leaving that area, the paired PIR sensor, which is located outside of that area, will detect and start to capture the movements. In this way, the tracking process will continue in the whole areas of a home by a network of PIR sensors.

The transmitted packets are collected by base node in a log file to be used for mobility pattern analysis based on their chronological order of their occurrence. For instance, the mobility pattern of a person can be deduced from the sequence of visiting areas by ordering the place of events based on their occurrence. Furthermore, the time duration in each area can be calculated by subtracting of last and first captured events in a set of successive events recorded by a same senor. In addition, the number of recorded events in each area can also be an indicator of the activity level of the resident between and in each space/room.

### 3.3. Sensor Deployment

The first step in testbed design is sensor deployment. To have a sensor in each individual area of the house, a number of options for the sensor layouts can be selected due to the house floorplan and the required areas for monitoring. Sensors can be mounted at different corners of each area attached to the wall or ceiling. Their effective range (approximately 10 m), the sensing point of view, plus their sensing angle will directly affect the data collection quality and the number of recorded events. The sensing angle depends on what type of sensor is chosen for installation, i.e., either using small area sensors (small angle—SA—with a less than 180° field of view) or wide area sensors (wide angle—WA—with detecting angle up to 360°) or even choosing no sensor placement. Since we chose to mount the sensors in a paired way to cover every entrance of the home and detect the transitions in our proposed solution, the number of possible layouts will be limited. In addition, the limitations caused by the building architecture and in some cases the furniture’s location, reduce the number of deployable layouts to fewer possibilities.

**Figure 2:**
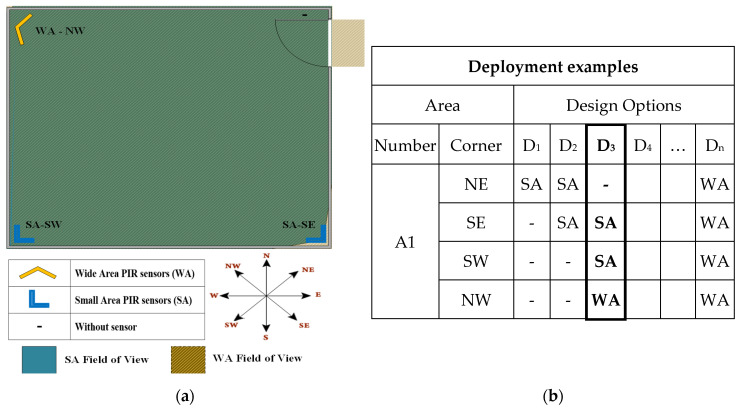
Design options for an example area with a door in NE corner: (**a**) an area with four corners, and (**b**) possible design options for sensor deployment.

A number of design options for a sample area with a door in NE corner is listed, e.g., D3 is illustrated in Figure 2. In this example, the door limitation would not allow a sensor to be placed in NE corner, therefore a sensor in SE can play the role. In this case, although a design option with a wide area PIR sensor mounted in NW corner or a place exactly in front of the door is possible, but it can lead in inaccurate transition detections when the door entrance is left open. Therefore, some of the design options can be eliminated in a preliminary phase.

**Figure 3:**
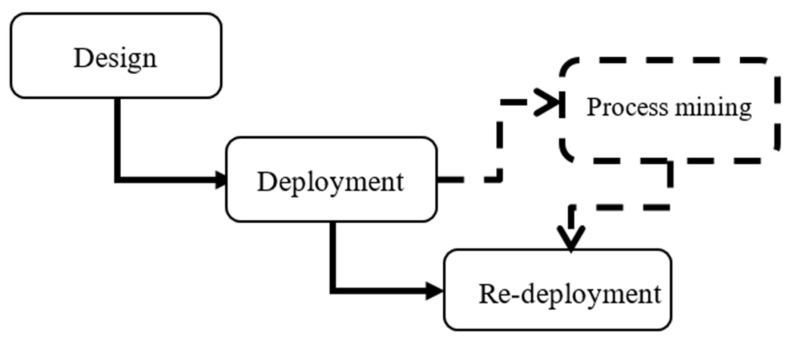
Proposed solution process using process mining using confusion matrix of transitions in order to change sensors’ position or angle.

The three conventional steps to set up a testbed is depicted in Figure 3 with the transition process highlighted in solid lines. Our proposed solution suggests improving the design with the help of process mining tool to help with the selection of the design options. We have experimented process mining for validation of our proposed solution. While the redesign process can be advanced to be operated automatically, in this paper, we have taken a number of examples manually to test the concept. Coverage overlaps between the sensors are inevitable in some cases which will lead to the recording of incorrect events such as redundant transitions between two areas or impossible transitions between two non-adjacent areas which cannot happen in the reality. Overlooking this issue can reduce the quality of the data collection and makes the results obtained from data analysis invalid. Therefore, the process mining and re-deployment steps can be used to identify the problematic areas of deployment and they can be repeated until achieving an appropriate design with an acceptable error rate during data collection (e.g., less than an acceptable threshold by a use case).

### 3.4. Testbed Implementation

**Figure 4:**
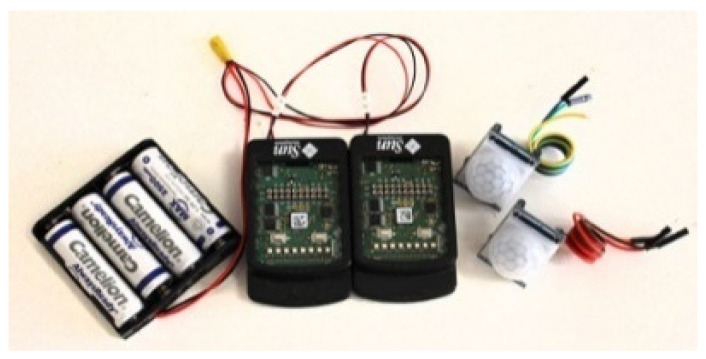
Observation unit includes a pair of passive infra-red (PIR) sensors, a SunSpot sensor, and a battery package.

Unlike the lab environment, change and improvement of a system after sensors installation, while people live in that place, would not be easily possible and can be challenging. Hence, the number of required sensors, their locations, and sensing angle should be designed and tested to cover every entrance and the whole space of the home based on the house map before the installation. To provide more reliability in PIR sensor data collection, we packed observation units for each doorway using a pair of PIR sensors, a SunSpot sensor and a battery package of four rechargeable AA batteries (Figure 4) and place these units on the border between two areas in a way to cover both areas as well as the border.

SunSpot sensors are programmable devices developed by Sun Microsystems which can be used to create a wireless sensor network. These devices communicate using IEEE 802.15.4 standard (also known as Zigbee) and their board included an ARM-based microprocessor, sensors such as accelerometer and analogue/digital I/O pins, which in this scenario are used to read PIRs state. The SunSpot nodes are directly connected to a sink which is responsible for gathering data and this provides a suitable wireless infrastructure for transmitting events captured by PIR sensors.

For a shared space between multiple area which has entrances to more than one room (for instance, a corridor), all doorway PIR nodes can be paired with the common space PIR to collect the movements in shared space with a single sensor. Further, the sink node should be placed in a spot that is accessible by each node over one hop wireless transmission (in order to eliminate the need for packet forwarding and routing algorithms).

## 4. Testbed Deployment and Evaluations

As mentioned earlier, deploying a smart home testbed requires a careful design based on the monitoring parameters required by the clinical staff. An accurate data collection in a residential allows the clinical team to rely on the collected pattern of the resident’s behaviour and to make necessary actions (such as prescribing a medication or rehabilitation therapy to prevent a predicted problem such as depression based on the translated symptoms, e.g., mobility pattern).

We examine issues related to data collection and changes in the topology in three phases, namely, deployment phase (primary testbed setup), post-deployment phase (testbed data validation and comparison), and re-deployment of additional nodes (secondary testbed) if required. Furthermore, behaviour assessments of the collected data are presented and discussed in Section 5 to provide a complete cycle of the monitoring service.

### 4.1. Deployment Phase

**Figure 5:**
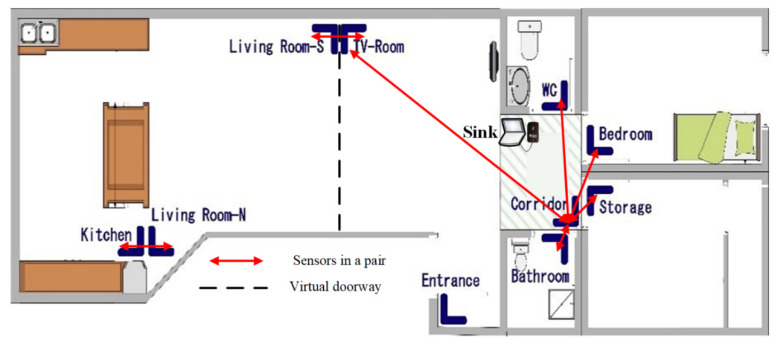
Positioning of PIR sensors and sink node in testbed for deployment phase.

The testbed that we are using to collect data is a two-bedroom flat with a single-resident which includes six doorways: main entrance, bedroom, storage (i.e., second bedroom in our charts), bathroom, kitchen, and WC. Moreover, to partition the movements in the living room and open-plan kitchen areas, additional PIR units are installed which form “virtual doorway”. The virtual doorway is highlighted in Figure 5 to separate the space between the “Kitchen” and “Living Room-N” as well as the “Living Room-S” and “TV-Room”. Further, the sink node placed approximately in the centre of the apartment to be within the radio coverage of all sensors. As an example of considering sensors as a pair, for instance, the “Kitchen” sensor and “Living Room-N” sensors illustrated in the Figure 5 are considered as a pair and every two consecutive event with the label of “Kitchen, Living Room-N” or “Living Room-N, Kitchen” identified as “leaving the kitchen” and “entering the kitchen” events, respectively.

During the deployment phase, we collected data over a course of 21 days by applying five observation units in the case-study home to monitor a solo-resident in the described two bedrooms flat using the PIR nodes.

**Figure 6:**
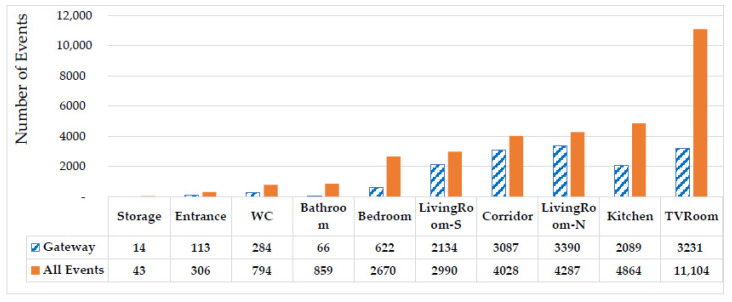
Comparison between all collected events and transitions in deployment phase.

The Figure 6 demonstrate a comparison between all events captured by sensor nodes in each space and the transitions. Transitions referred to the movements detected by a pair of nodes, which reflects moving from an area to the adjacent area and can be used to track the mobility pattern of the resident and the activity levels in each space. The larger number for all events at TVRoom relative to the number of transitions made at this area indicates that the residents had more movements and activities there without leaving the area while the ratio of transitions to all events at LivingRoom-N is higher and shows resident mostly used this area to reach other areas of the apartment.

### 4.2. Post-Deployment Phase-Data Validation of Deployment Phase

By considering the physical layout of the building and assuming that the case study subject follows a logical pathway between areas, jumping between areas will be impossible; hence, all captured events must follow logical sequences based on the building floorplan. In this way, the applied pairing setup can provide the required sequence of events in order to assist the validation process of collected data. For instance, transitions between the Bathroom to the WC and the storage to the TV-room are not physically possible due to the floor plan as shown in Figure 5. Furthermore, the resident notes can assist us for overall validation of the collected data. Using the process mining tool PMApp [41] and the PALIA algorithm [40] it is possible to create a view of the whole process, an aggregated model of the resident pathway during the studied period.

**Figure 7:**
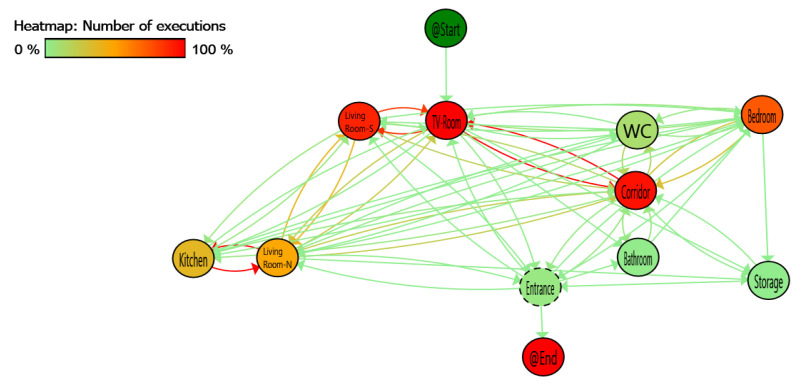
Deployment phase process mining data analysis: A colour heat map is applied based on the number of executions, red shows the most executed and green less executed.

We can see the process mining discovery result of PALIA in Figure 7 represented as a TPA. Each node of the graph represents a location and the arrows the transition between locations. Applying a heat map over the TPA based on the number of times a node or transition is executed, the elements vary from green to red, red being the most detected node or transition. It is necessary to remark that before discovery, all the consecutive detections at the same place has been converted to one detection in that place with long duration.

Based on the explained heat map, some red nodes are highlighted as the places in the dataset with the highest number of detections and in the same way the red transitions show between which locations more movements were detected. The sensors between Living Room-S and TV-Room as well as Corridor space to the TV-Room are the most active. Although this is consistent with the reported user behaviour, the high number of detections and transitions between nearby locations shows a possible sensor overlap problem.

The TPA model also shows that there are impossible transitions between non-adjacent locations. To quantify these errors, the data used for the TPA has been structured as a confusion matrix of transitions shown in Table 1. As it can be observed the false positive data highlighted in colour-filled cells with red colour result in inaccurate data based on not physical possible transitions between locations, which requires a re-examination of the testbed setup. Minimum incorrect detections (such as this type of wrong detections) are an indicator of a good placement of the sensors in a location system.

Based on the post-deployment analysis, to overcome the observations made by the analysis of the deployment phase, reviewing the TPA and the confusion matrix of transitions several conclusions can be obtained:

1. On 21-day dataset configuration, most of the problems are derived from the interaction of Kitchen and Living Room-S, Living Room-N and TV-Room with corridor sensors. Separation of larger areas by virtual gateways is problematic and limits the accuracy of the mobility pattern detection. This is due to the physical closeness of the deployed PIR nodes lacking a wall as an obstacle which may lead to misdetections of such transitions.
2. Between Bedroom and TV-Room there is a specific problem that affects these sensors due to the overlapping angle of view of the sensors. An overlap occurs when a sensor in one area detects a movement within another area (out of the area covered by that sensor and supposed to be covered by another sensor), this leads to an overlapping detection and will create incorrect event.

Once upon post deployment and data validation by process mining finished, if the desired accuracy and quality in data collection was not met, the next step will be a re-deployment phase as illustrated in Figure 3. According to the post deployment analysis and its conclusions, a re-deployment phase is set up and reported in the following subsection.

### 4.3. Re-Deployment

**Figure 8:**
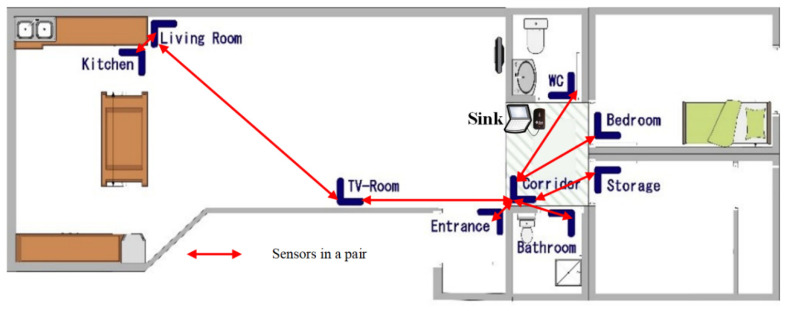
Positioning of PIR sensors and sink node in testbed for re-deployment phase.

The testbed layout is redesigned to minimise the inaccurate data collected in the deployment phase and data is collected in a similar condition over 70 days (based on resident’s report). The testbed layout is modified (as shown in Figure 8) where the positioning of the sensors that capture the movements in the kitchen and living room as well as main-entrance are updated as followings:

1. Field of view of the v-gateways are limited to avoid misdetection of the case study in the adjacent areas.
2. The positioning of the corridor, entrance, living room, kitchen, and TV-room sensors are changed to avoid the overlapping detection.

**Figure 9:**
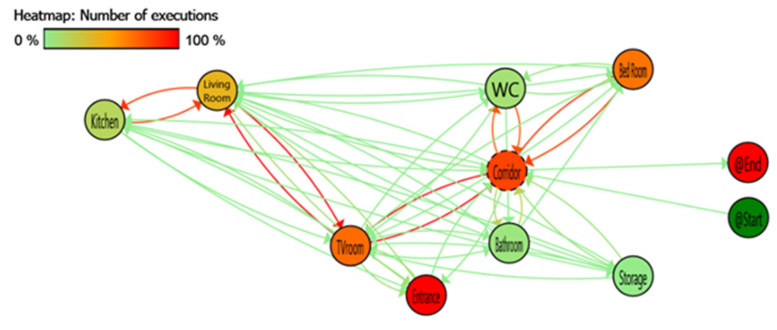
Re-deployment phase process mining data analysis.

As in the previous deployment, the raw data is analysed using PMApp. The TPA for re-deployment testbed setup is represented in Figure 9 and the confusion matrix of transitions on Table 2.

This time the errors are detected as done in deployment phase between adjacent areas or when we aim to split large size areas without physical obstacles between areas such as walls. With the new design, we observed the following issues:

1. Main problems are between Entrance and Living Room which sensors are in the same line of vision without physical obstacles.
2. With a reduced error values in relation of previous point, movement detections between the Bedroom and the WC suffers from errors. It can be related to that the movement between the two adjacent locations does not allow the Corridor sensor to detect the transition.

To compare both deployments, we need independent evaluation indicators (or metrics) from the length of the dataset (as we have 21 days of data in deployment and 70 days in the re-deployment phase) which should be independent of the number of detections in the dataset. Two complementary indicators have been defined based on the confusion matrix of transitions.

The first indicator is the erroneous transition probability indicator (ETPI). This indicator provides information of which is the percentage of the transitions that are erroneous. This indicator is obtained by dividing the total number of erroneous transitions by the total number of transitions. This indicator allows comparing both deploys to see which of both has more quantity of errors about the total number of transitions. The reliability of this indicator can be affected if two adjacent locations have a big number of transitions between them. For example, TV-Room and Corridor in the re-deployment design. To mitigate this problem, we have defined the second indicator total transitions per hour indicator (TTPHI). Which indicates how many transitions by hour we have, so we can see how many activities we have in the dataset independent of the duration. The indicator is calculated by dividing the number of transitions at the dataset by the hours of measurement.

Using these two indicators if both indicators decrease, we detect an improvement on the re-deployment. If only the ETPI decreases, we have also an improvement, and for the remaining combinations, we cannot conclude that we have an improvement, a detailed manual revision of the data is needed in those cases.

When we compare both deployments, we see at Table 3 that ETPI and TTPHI both decrease notably, hence, for the new design improvement. The error rate yields and improved accuracy of 81.57% to 95.53% in re-deployment phase as a result of applying the PALIA process mining algorithm.

### 4.4. Comparison with Existing Datasets

To validate our results, our testbed is compared with a publicly available dataset from Washington State University by Cook et al. project [26]. In CASAS project, various sensors (such as motion sensors, contact switch sensors and digital temperature sensors) are used to perceive the status of residents and their surroundings. We have selected one of them which is a common apartment enhanced with passive infra-red (PIR) presence sensors based on a similar floor plan and number of PIR nodes to our testbed, a two-bedroom apartment (dataset number 46, testbed name HH124 in CASAS project) to analyse data in order to compare their collected data with our testbed data. This comparison helps us to validate our testbed.

Installed sensors in CASAS testbed are placed to strategically track all the movements and activities of the inhabitant inside different rooms of the house (including kitchen, living room, bathroom, dining room, bedrooms, and corridor). The sensors used in specific locations of the building such as ceiling to cover the whole area of places or on top of devices like refrigerator as well as bed and chairs in the living room [45].

**Figure 10:**
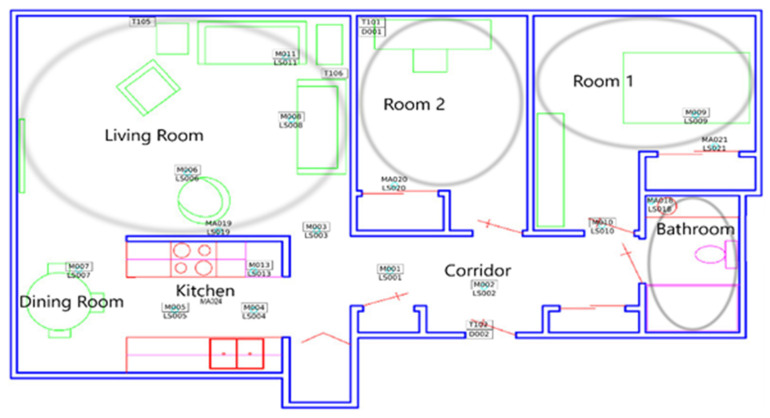
CASAS testbed (HH124) distribution.

To validate our results, we have applied the same approach to the CASAS-HH124 dataset. At Figure 10, we can see the distribution of the house and the sensors. There are some wide-area PIR sensors marked with the prefix MA and small-area PIR sensors marked with the prefix M. Using PMApp, we have imported the dataset and generated the TPA, confusion matrix of transitions and the two defined comparison indicators.

**Figure 11:**
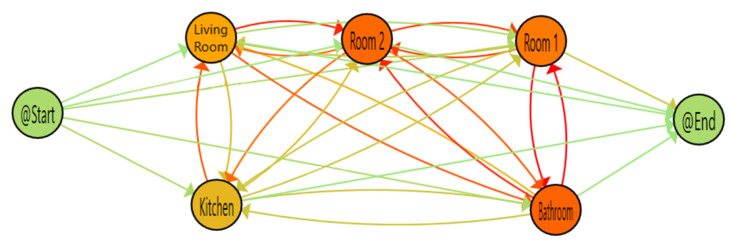
Parallel activity log inference algorithm (PALIA) timed parallel automaton (TPA) for CASAS using wide-area sensors.

For the first part of evaluation, we have used only wide-area sensors which are similar to our deployment case. We show the results of this study at Figure 11 and Table 4, using physical distribution as a reference there are no detection errors on the dataset. However, if we analyse the data, we can see that all wide-area sensors have physical barriers between them, so that avoid PIR inherent location interferences. In other word, without considering corridor as the only point of connection for different areas by not using sensor in this area, all the transitions in home could happened logically, so they are labelled as valid and detection error will be equal to zero. In addition, restricting the sensor placement to the areas which are separated by physical barriers (e.g., walls) may result in some indistinguishable and without sensor spaces which may not be suitable for some applications and studies. However, adding sensors to these areas, as we mention in previous conclusions, could be challenging because sensors without physical barriers are the most problematic. To further investigate, the installed small-area sensors in conflictive areas are considered as well, such as Corridor that interconnects all the areas or Dining Room which is part of a big area in common with Kitchen.

In some studies, or scenarios, avoiding conflictive location areas, can be useful and do not affect the study. However, not all the houses follow a similar layout to allow this. Furthermore, in some studies, avoided areas can affect the results. For that reason, we decide to include some small-area sensors to flag such areas and compare with our results.

The included sensors in the next part of evaluation are M007 as Dining Room area, M001 as Corridor1 area and M002 as Corridor2 area. Both corridor sensors can be combined in one, but we decide to maintain separate to simulate a study that use it to detect directionality of resident movements.

**Figure 12:**
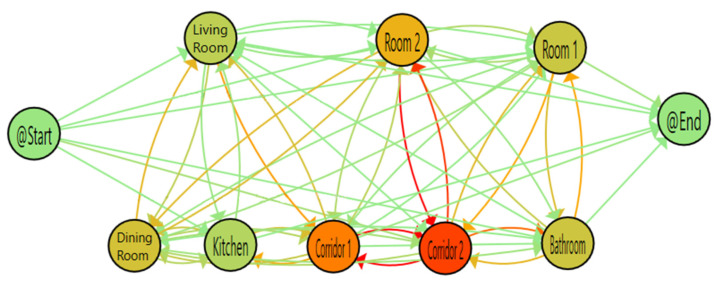
PALIA TPA for CASAS using additional sensors.

After the changes of the sensors and areas included, we obtain the TPA and the confusion matrix of transitions of Figure 12 and Table 5 were we can see some results that validate the previous conclusions obtained with our dataset, all related to the conflictive areas.

1. Corridor areas are the ones with a greater number of detections and with a lot of interchanges between them, because they are in a shared area without any physical barrier. These detections are not categorised as an erroneous based on the physical structure of the house but introduce an extra increment on the number of detections.
2. Transitions between Rooms and Bathroom are not well detected by Corridor sensors. Like in the Corridor area (in re-deployment testbed), the resident can change quickly from these adjacent areas and the sensor not always capture it.
3. Dining Room has a lot of error interactions with Corridor sensors since they are in the same shared area.
4. Furthermore, a none expected behaviour detected is the elevated number of errors between Dining Room and Room 2, being that there are at least two sensors in the middle. A deep review of the resident pathway shows that these transitions occur after some hours or days. With the process mining tool PMApp we can detect the case, but extra information from CASAS team will be necessary to explain that behaviour.

Finally, to validate the two defined comparing indicators ETPI and TTPHI we have calculated both in three different designs based on which sensor or areas has been selected:

1. Design that only considers wide-area PIR sensors.
2. Design that includes previous areas, M001 as Corridor 1 and M002 as Corridor 2.
3. Design that considers design-b sensors and M007 sensor to include the Dining Room area.

We show the results of comparing indicator on Table 6 and we can see that as we include conflictive detection areas both indicators increase, detecting the deterioration on the dataset information.

### 4.5. Recommendations for PIR Sensor Deployment

According to our experiences from the conducted analysis on the collected datasets from two implemented testbeds and also CASAS dataset, we have learned a number of lessons to share which can be used in the future smart home designs with PIR sensors to increase the efficiency of data collection and reduce detection errors and required time for system setup:

1. Based on our results, it is feasible to install PIR sensors in smart homes in order to collect data about resident activities (in a solo-resident house) and the mobility pattern could be extracted from collected data.
2. For efficient installation, the whole space of a home should be divided to different areas and a PIR sensor should be placed in each one.
3. It is better to mount PIR sensors in pairs and install them in doorways so that the sensors of each pair could detect entering and leaving time of an area based on captured events sequence, in addition to covering the movements within the corresponding area of each one of them.
4. For common spaces (e.g., “Corridor”) with an entrance to more than one room, one PIR sensor in common space can play a role of pair node for all PIRs in adjacent rooms’ doorways and collect the actions over doorways to the common space. This will reduce the number of required sensors and can also lead to fewer number of captured events due to removing the redundancy of covering a common space by multiple sensors.
5. PIR sensors should be mounted in a way to not add overlapping areas between their sensing range or minimise it as much as possible. In the case of existence of an overlap between two covered spaces of two sensors, by moving the resident within overlapped area, both sensors detect the movement which result in redundant captures and events and subsequently unrealistic higher value of TTPHI. Moreover, if the overlapping area caused by two sensors within non-adjacent area (similar to the 21-day testbed), such transitions detected as erroneous transitions which decreased the accuracy of the system (i.e., increase in ETPI value). Hence, it is better to limit the associated space to each sensor to physical barriers such as walls and restrict the transition space between two areas to doorways. This will prevent capturing unnecessary transitions for overlapping areas and make the number of captured transitions more accurate.
6. Separation of large areas by creating virtual gateways with paired sensors and open large spaces, if not handled in an appropriate way, could be problematic and increased error percentage.
7. Further, it should be noted that restricting the use of sensors to areas which are separated by walls and ignoring the sensor installation in common or large places may result in uncovered areas as well as some undetected transitions. This approach may not be acceptable for some applications which require mobility pattern detection with higher accuracy. In such cases, the designer of system can decide about the installation of sensors in these locations based on the expected goal of the system.
8. It is possible to remove the overlapping areas of the sensors by limiting their coverage angles, by using small-area instead of wide-area PIR sensors or by changing the point of view of each sensor (for instance sensor could be attached in a location near the entrance facing to the opposite side wall or even attached to the ceiling. Like what we did by changing the location and point of view of entrance sensor in re-deployment testbed).
9. Each sensor has limitations in sampling rate and detection (usually depends on hardware characteristics). If a sensor does not have enough time to detect a transition between adjacent areas, there is possibility that it will not always be able to capture the transitions due to non-adherence of these limitations (similar to the re-deployment testbed case between Bedroom and WC, as described in Section 4.3). After discovering the origins of errors caused by hardware limitation, solutions such as changing the sensor field of view or using high sampling rate sensor types can be used in redeployment to resolve the problem.
10. For some specific activities, such as cooking and sleeping, access to their detail (time spent on the activity or start and end time of the activity) can provide better information about the behaviour pattern. For this purpose, dedicated sensors in the location of performing the activities (for instance on top of the bed or stove) need to be installed.

## 5. Behaviour Assessment

Behaviour changes are consistent with the changes in cognitive and physical health and continuous sensor data collection and using technology assistance in everyday can help to observe these changes which in some cases might be difficult to detect. These changes can be too gradual or too subtle like changes in time spent on key activities which researchers have found it as a sign of dementia in early stage [46].

The ambient data or specifically smart home data consists of raw sensor-data streams along with the events’ timestamp, the ID of capturing sensors and type of events. To be able to interpret the raw sensor-data streams, day-level activity features can be used to represent activity-level, and behaviour information for each subject. Then, the extracted behavioural information can be used to analyse behaviour patterns. In addition, the longitudinal monitoring of behavioural information can be used to monitor older adults’ health state and assist in the assessment of some age-related diseases and disorders progress. For this purpose, the extracted day, week, month, or even year level activity feature vectors during a long period of data collection can be used with behavioural detection change algorithms to assess the functional health of elderlies [45,47].

We conducted a review on extractable features from smart home data in the literature to interpret activities and analyse behaviour patterns. Finally, a list of day-level activity features which can be extracted from our testbed dataset is selected to use for behaviour assessment (Table 7) and to show the ability of our smart home system model for behaviour assessment, a number of parameters which could reflect the activity level are reported in the following of this section.

### 5.1. Number of Events per Day

**Figure 13:**
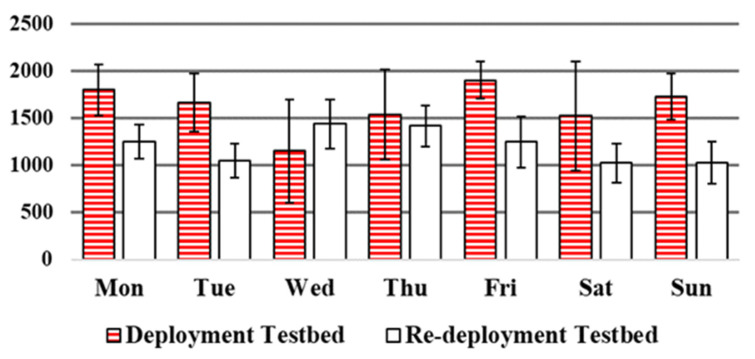
Average number of events. comparison between our deployment and re-deployment datasets.

The number of sensor activation or events is the first metric which calculated for comparison of our three datasets. A packet will generate and transmit in exchange for any change in every sensor state and the total number of transmitted packets per day can present the activity level of the resident. Comparison on these three datasets, based on weekdays, leads us to Figure 13. As it is obvious from the chart, almost the largest number of captured events by sensors occurred on weekends (Saturday and Sunday for CASAS dataset and since weekends in Iran is Thursday and Friday, these days for the other two datasets). In addition, in relative to weekdays, due to the error bars these days also have more deviations indicate that the behaviour of resident is not similar in all the weekends and in some weeks the person spent more hours outside the home while in other weeks had spent the whole day at home which is matched with the reports.

**Figure 14:**
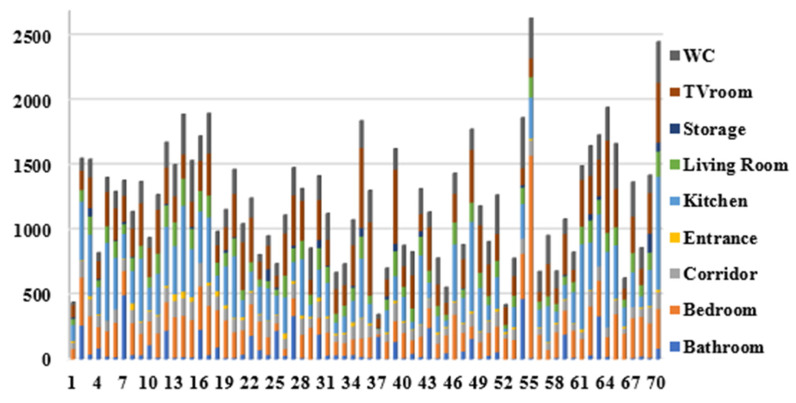
The total number of captured events per location for re-deployment testbed dataset.

In addition, the number of captured events per location can reflect how much of subject’s activities are assigned to perform a specific task such as personal hygiene activities, cooking, and eating. Figure 14 shows the total number of captured events per location for our 70-day testbed.

### 5.2. The Number of Captured Events and Total Covered Distance

**Figure 15:**
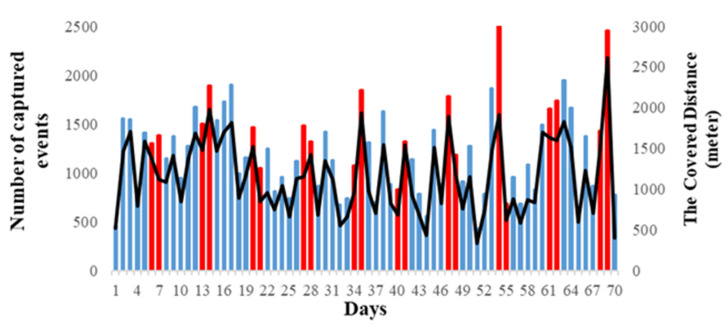
The total number of captured events and total covered distance per day for re-deployment testbed dataset.

The inhabitant’s daily covered distance can be estimated by knowing the apartment’s floorplan and the placement of sensors within the apartment. The dimensions of the rooms, distances between the centre of each area with the next one and distances between sensors are calculated accurately. Then, for every pair of events captured by a single sensor, two points are selected randomly within that area or two points are selected from two adjacent areas for every captured transition and then the distance between these two points is calculated. Adding all of the calculated distances throughout the day provides an approximation of the daily total covered distance. Note that this approach does not consider the existence of walls or other obstacles in the path of movements and assumed the resident walked in a straight line from the area covered by one sensor to another area covered with different sensor. Figure 15 presents the covered distance and the number of captured events for each day in re-deployment testbed dataset. The red bars indicate the weekend days; our subject is more active over the weekends as spending more time at home.

### 5.3. Activity Pattern

The activity pattern collection helps with behavioural assessment of the data. For instance, it can show the time spent outside home for work or social interaction depending on the time of the day and being weekdays or weekends which can reflects on the mood, level of loneliness, and cognitive health of the house single resident [6]. The deployment (collected over 21 days) data highlights the fact that an average of 29 percent of the time the resident is out of home on every day.

**Figure 16:**
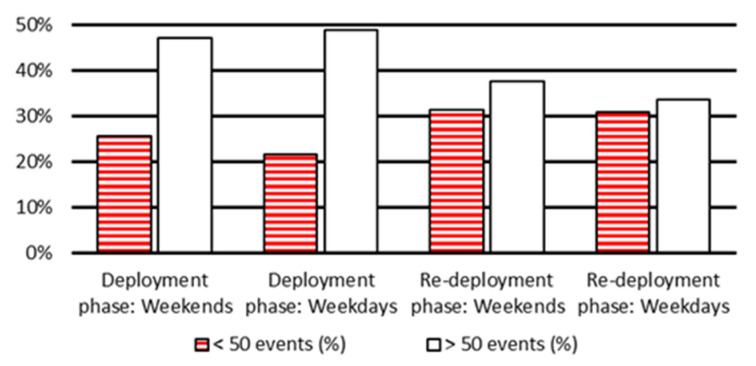
The average percentage of hours in a day for three activity levels of more than 50, less than 50, and no event for deployment and re-deployment testbed during weekdays and weekends.

In addition, considering an average of 50 events per hour as a minimum activity level for a healthy adult, the percentage of activities can report a pattern of the resident’s activity. An increase in the low activity level percentage can be taken as an early sign of depression [48]. Figure 16 shows the average percentage of hours in a day for three activity levels for deployment and re-deployment (collected over 70-day) dataset during weekdays and weekends. Three activity levels labelled with “no event”, “<50 events” and “>50 events” which show times being out of home, hours with low activity level, and high activity level in an hour, respectively.

Figure 16 shows the resident activity volume with “no event” to be the case of being out of home, “<50 events per hours” for a low activity and sedentary state (such as sleeping, studying, and watching TV), and “>50 events per hour” for a high activity level (such as taking shower, cooking, washing dishes, and house cleaning). Results show that the total number of “<50 events per hour” for both weekdays and weekends are recorded more in the re-deployment compared to the deployment phase due to higher precision of event detection as a result of lower number of sensors and reduced overlapping coverage areas—this can match results presented in Table 1 and Table 2—“>50 events per hour” is recorded less as a complementary to the increase of the low event record registration.

### 5.4. Time Duration Spent in Each Location

**Figure 17:**
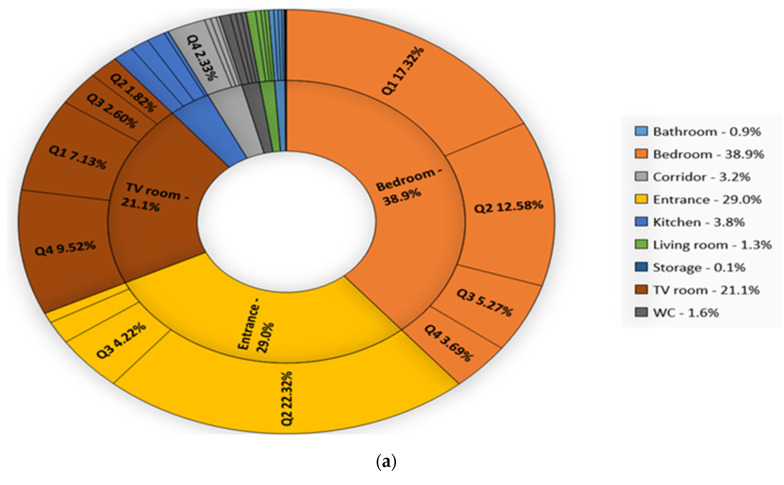

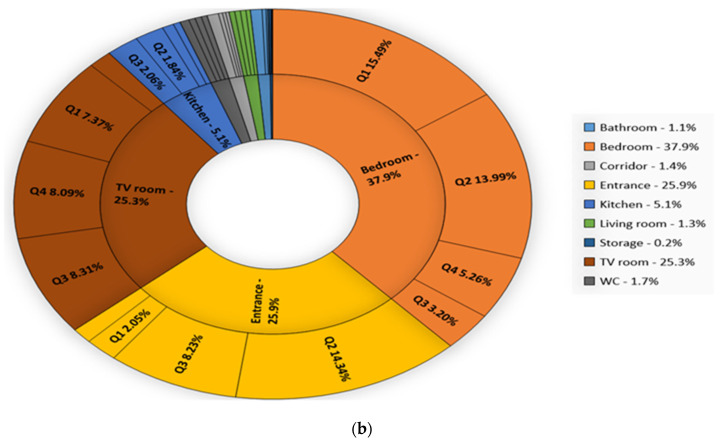
The average time duration in each location in a day and in different quarters of a day for (**a**) weekdays (**b**) and weekends in re-deployment dataset.

The time duration spent in every location of a home and in various times of a day can be linked to different tasks and activities. Hence, we compute this amount of time for the collected 70-days dataset and calculate the average time spent in each location for both weekdays and weekends. In addition, we split a day into four time periods: Q1 from 00:00 to 05:59, Q2 from 06:00 to 11:59, Q3 from 12:00 to 17:59, and Q4 from 18:00 to 23:59, and calculate the average time spent in each quarter. The results presented in Figure 17, the home resident spent 38% and 39% of his/her time in the bedroom for sleeping and studying in weekends and weekdays, respectively. Furthermore, we observe second highest duration recorded at the entrance point which represents the time the resident being out of home for each day (mainly in Q2). The resident stays out of home 3.1% more in weekdays compared to weekends and 21.3% of this increase is happened in Q2 time interval including working hours and almost 2% decrease happened in being out of home within Q4 time interval for weekdays. This shows that the resident tends to go stay home after working hours in weekdays. Furthermore, the residents watch TV 4.2% more in weekends, a third of which is in Q3 period (i.e., typical working hour in weekdays).

### 5.5. Behavioural Assessment Usage

Using sensor systems in the home environment will help continually monitoring of resident activity inside the apartment, which is one of the most valuable and needed functionalities of the smart home technology. These sensory systems could be used as an intervention tool to influence clinical outcomes by identifying an individual typical mobility pattern, then recognising when the pattern changes. The quantity and objectivity of two or more periods of data, or windows, can be compared to track different types of changes. If the comparison shows a considerable difference between the data of two selected time interval, this can be interpreted as a significant behaviour change and requires further investigation.

Two forms of pattern deviation are possible, first a sudden change because of a specific health event and second a gradual change as a result of a deteriorating situation [27]. In both cases, catching declining conditions by taking the advantages of smart homes capabilities could be faster than using traditional healthcare practices. Providing this information about the type of detected change and its magnitude in the form of reports can be delivered to the medical team to help them in better understanding of patient status. In addition, access to this information as feedback will motivate and encourage older adults to change their behaviour in an effort to create a healthier lifestyle and empower them to take an active role in their own health.

However, despite all these benefits, there is still a long way to achieve the maximum productivity of smart homes. Annotating the sensor data in order to find a relationship between sensor detected events with sentinel health events remains as a great challenge. Therefore, further investigation to find connection between sensor data and health events is required.

## 6. Conclusions

Considering the increasing aging population, in this work, we focus on smart home mobility pattern monitoring system to prolong independent living of solo-residents. We discussed the wireless portable sensors which enable temporary elderly monitoring systems, e.g., for post hospital discharge period and designed a testbed by using PIR sensors. We set up a testbed to evaluate our data collection platform and compared it with existing CASAS project dataset. In addition, the testbed setup phases are described, i.e., a deployment setup (i.e., 21 days), a post-deployment analysis, and a re-deployment setup (i.e., 70 days).

The main contribution of this work is to apply intelligence from process mining techniques to improve the re-deployment process more accurately. We have used a process mining algorithm (PALIA) and PMApp to validate the accuracy of the collected data in post-deployment and the insights are used in the re-deployment phase. Accuracy analysis of data collection experiments led to reduce the error rate from 18.43% to 4.12%, respectively, in the post-deployment phase. Furthermore, we apply our proposed approach to one of the CASAS datasets and get similar results which shows the effectiveness of our analysis method to be used as a generic approach using other sensing technologies.

Finally, we show the collected data can be further processed to map the level of activity of the solo-resident for behaviour assessment, prognostic disease, and prevention. For this purpose, a series of analysis about behaviour assessment by considering day-level activity features presented to show the possibility of acquiring behaviour pattern of a resident in a smart home. This pattern could be evaluated to detect possible changes as a result of deviations in cognitive and physical health. Early detection of changes in behaviour with the assistance of technology will improve the clinical health interventions and provide more awareness about unhealthy lifestyle to older people.

## 7. Future Directions

Our proposed approach of optimising a setup utilises information obtained from process mining techniques and can be considered as a generic approach in order to detect and correct the design flaws of a monitoring or tracking system.

The deployed smart home testbed setup in this work is designed to monitor elderly aftercare condition using PIR sensors to collect data about the resident mobility pattern. While applying low cost PIR sensors can provide sufficient information for medical assessment, the solution can be enriched by capturing additional ambient information such as temperature, light, RFID tags, utility usage, radio signal strength, and ultrasonic sensors.

Mobility pattern detection and assessment methods can be applied in other sectors, such as industrial tracking of products in warehouses [49], smart tracking/monitoring for smart cities, person tracking in a building (e.g., workers in a factory or nurses in a hospital [40] to improve the working efficiency), as well as customer tracking in shopping malls [41]) for marketing and advertisements, which indicate industrial relevance for further research and development.

## Figures and Tables

**Table 1:** Confusion matrix of transitions in deployment phase: Transitions between non-adjacent locations are filled in red as false detections.

	Bathroom	Bedroom	Corridor	Entrance	Kitchen	Living Room-N	Living Room-S	Storage	TV-Room	WC
**Bathroom**	0	3	92	0	0	0	0	0	0	0
**Bedroom**	0	0	610	3	4	6	4	1	101	75
**Corridor**	89	551	0	64	90	423	314	16	2073	287
**Entrance**	1	6	78	0	0	5	5	0	19	0
**Kitchen**	0	4	56	0	0	2362	88	0	32	1
**Living Room-N**	0	39	422	9	2318	0	740	1	452	2
**Living Room-S**	0	8	375	2	99	732	0	0	1549	0
**Storage**	0	0	18	1	0	0	0	0	0	0
**TV-Room**	5	131	1948	36	32	454	1613	1	0	9
**WC**	0	62	308	0	0	1	1	0	2	0

**Table 2:** Confusion matrix of transitions in re-deployment phase. Transitions between non-adjacent locations have been marked in red as false detections.

	Bathroom	Bedroom	Corridor	Entrance	Kitchen	Living Room	Storage	TV-Room	WC
**Bathroom**	0	1	245	0	0	1	1	4	0
**Bedroom**	0	0	1009	0	0	2	0	22	52
**Corridor**	246	1029	0	26	5	14	78	1262	903
**Entrance**	0	0	18	0	9	102	0	122	0
**Kitchen**	0	0	6	6	0	1023	10	21	0
**Living Room**	3	2	34	117	1034	0	3	1240	1
**Storage**	0	0	79	0	11	2	0	2	0
**TV-room**	2	5	1263	102	7	1288	2	0	8
**WC**	1	48	909	0	0	2	0	4	0

**Table 3:** Comparison of applied evaluation indicators.

Deployment	Erroneous Transition Probability Indicator	Total Transition Per Hour Indicator
First design	18.43%	31.8256
Second design	4.47%	6.1334

**Table 4:** Confusion matrix of transitions for CASAS using wide-area sensors.

	Bathroom	Kitchen	Living Room	Room 1	Room 2
**Bathroom**	0	4	6	21	13
**Kitchen**	4	0	11	4	5
**Living Room**	11	5	0	2	13
**Room 1**	17	4	4	0	12
**Room 2**	11	10	10	12	0

**Table 5:** Confusion matrix of transitions for CASAS using extra sensors.

	Bathroom	Corridor 1	Corridor 2	Dining Room	Kitchen	Living Room	Room 1	Room 2
**Bathroom**	0	0	16	1	0	1	20	10
**Corridor 1**	1	0	56	10	19	13	1	8
**Corridor 2**	28	59	0	6	0	0	15	35
**Dining Room**	3	7	4	0	7	16	3	14
**Kitchen**	0	14	0	11	0	3	0	0
**Living Room**	0	22	1	9	2	0	1	1
**Room 1**	14	2	20	2	0	1	0	5
**Room 2**	2	6	42	15	0	2	6	0

**Table 6:** Comparison of three different designs of CASAS based on two evaluation indicators.

Design	Erroneous Transition Probability Indicator	Total Transition Per Hour Indicator
(a)	0.00%	0.1228
(b)	19.29%	0.3149
(c)	27.54%	0.3664

**Table 7:** Day-level activity features applied in our study (revised from [45]).

Features	Types
The total number of events (sensor activation) per day	Mobility-related features
Total distance covered walking inside the apartment per day	
Time spent per day in being out of home	Duration of specific activities
